# The Troll Is Weakened but Not yet Defeated: An Update on Cytomegalovirus Management in Transplantation From the International CMV Symposium 2025

**DOI:** 10.1111/tid.70223

**Published:** 2026-05-05

**Authors:** Camille N. Kotton, Martina Sester, Julian Torre‐Cisneros, The International CMV Symposium Faculty

**Affiliations:** ^1^ Transplant and Immunocompromised Host Infectious Diseases Infectious Diseases Division Massachusetts General Hospital Harvard Medical School Boston Massachusetts USA; ^2^ Department of Transplant and Infection Immunology Saarland University Campus Homburg Homburg Germany; ^3^ Center for Gender‐specific Biology and Medicine (CGBM) Saarland University Homburg Germany; ^4^ Service of Infectious Diseases Reina Sofia University Hospital Maimónides Institute for Biomedical Research of Córdoba (IMIBIC) University of Córdoba (UCO) Córdoba Spain; ^5^ Centro de Investigación Biomédica en Red de Enfermedades Infecciosas (CIBERINFEC) CIBER de Enfermedades Infecciosas Instituto de Salud Carlos III (ISCIII) Madrid Spain

**Keywords:** cell‐mediated immunity, CMV immunoglobulin, cytomegalovirus, guidelines, hematopoietic stem cell transplantation, solid organ transplantation, vaccines

## Abstract

The 2025 International CMV Symposium convened in Königstein, Germany, bringing together transplant clinicians and researchers to address cytomegalovirus management following the publication of three major guidelines in 2025. The Transplantation Society, European Conference on Infections in Leukaemia, and American Society for Transplantation and Cellular Therapy each released updated recommendations reflecting significant advances, including letermovir prophylaxis for hematopoietic stem cell and high‐risk renal transplantation, maribavir for resistant or refractory disease, and cell‐mediated immunity testing to guide personalized prevention strategies. This review synthesizes symposium discussions, emphasizing translation of evidence‐based recommendations into clinical practice across diverse healthcare settings. Key topics included risk‐stratified prevention approaches balancing efficacy with practical implementation, precision medicine through immune biomarkers, adoptive cellular therapies advancing from experimental to clinical platforms, and vaccine development showing renewed promise after decades of setbacks. Symposium faculty highlighted persistent challenges despite recent progress, such as late‐onset disease after extended prophylaxis, economic barriers limiting access to novel therapies and diagnostics, resistance patterns evolving with increasing antiviral use, and infrastructure requirements for preemptive strategies that may be prohibitive in many settings. Case presentations illustrated real‐world practical challenges to effective guideline implementation. The integration of solid organ and hematopoietic stem cell transplant expertise enabled cross‐disciplinary learning while respecting population‐specific differences. As the transplant community advances toward defeating the “troll of transplantation,” continued therapeutic innovation must be coupled with implementation science, economic analyses, and global cooperation to ensure all transplant recipients benefit from these advances.

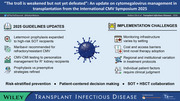

## Graphical abstract

This review synthesizes discussions from the 2025 International CMV Symposium on translating three recently updated international guidelines into clinical practice across solid organ and hematopoietic stem cell transplantation, highlighting advances in risk‐stratified prevention, emerging therapies, immune monitoring, and persistent implementation challenges.

**Abbreviations**
ALC: absolute lymphocyte count
allo‐HSCT: allogeneic hematopoietic stem cell transplantation
AML: acute myeloid leukemia
ASTCT: American Society for Transplantation and Cellular Therapy
BAL: bronchoalveolar lavage
BCMA: B‐cell maturation antigen
CAR‐T: chimeric antigen receptor T‐cell therapy
CHARMD: Comprehensive Herpesviruses Antiviral Drug Resistance Mutation Database
CMI: cell‐mediated immunity
CMV: cytomegalovirus
CMVIG: cytomegalovirus immunoglobulin
CMV‐QNAT: cytomegalovirus quantitative nucleic acid testing
CNS: central nervous system
CYP: cytochrome P450
D: donor
DSA: donor‐specific antibodies
EC_50_
: half maximal effective concentration
ECIL: European Conference on Infections in Leukaemia
ESCMID: European Society of Clinical Microbiology and Infectious Diseases
FOS: foscarnet
gB: glycoprotein B
GCV: ganciclovir
GRADE: Grading of Recommendations Assessment, Development and Evaluation
GVHD: graft‐versus‐host disease
HLA: human leukocyte antigen
HSCT: hematopoietic stem cell transplantation
IgG: immunoglobulin G
IPF: idiopathic pulmonary fibrosis
IV: intravenous
IVIG: intravenous immunoglobulin
LET: letermovir
MBV: maribavir
MMF: mycophenolate mofetil
MVA: Modified Vaccinia Ankara
NK: natural killer
PCR: polymerase chain reaction
PET: preemptive therapy
R: recipient
R/R: refractory/resistant
SOT: solid organ transplantation
TDM: therapeutic drug monitoring
TTV: Torque teno virus
VGCV: valganciclovir
VL: viral load
VST: virus‐specific T cell
WHO: World Health Organization

## 1 Introduction

Cytomegalovirus (CMV) remains the most significant cause of infectious complications following solid organ and hematopoietic stem cell transplantation (HSCT). Yet, there has been remarkable progress in managing this persistent virus in recent years, reflected in the publication of three major guidelines in 2025. The Transplantation Society published a new guideline on the management of CMV in solid organ transplantation (SOT) [1], while the European Conference on Infections in Leukaemia (ECIL) [2] and the American Society for Transplantation and Cellular Therapy (ASTCT) [3] published guidelines on CMV in HSCT (Table 1). Together, these guidelines reflect significant advances in research on new treatments and surveillance approaches, while also amassing high‐quality evidence to support recommendations previously considered conditional. Noteworthy updates include the establishment of letermovir for prophylaxis in HSCT and expanded use in high‐risk renal transplant recipients, maribavir for the treatment of refractory/resistant CMV, and endorsement of cell‐mediated immunity (CMI) testing for immunoguided prevention in clinical practice. Updates are also included for combination therapies, including CMV immunoglobulin (CMVIG) for refractory/resistant infection, and expanded guidance on treating pediatric patients.

**TABLE 1:** Key updates in 2025 CMV guidelines.

**Topic**	**SOT guideline** [1]	**ECIL** [2] **and ASTCT** [3] **guidelines**	**Strength of recommendation** ^a^
Diagnostics	Greater emphasis on CMV‐QNAT calibrated to the WHO standard	CMV serology recommended pre‐HSCT; pretransplant CMV PCR recommended	Strong (SOT); Moderate (HSCT)
Prevention: prophylaxis	Letermovir recommended in D+/R− kidney, investigational in non‐kidney transplants; PET option for D+/R− liver	Letermovir standard prophylaxis for R+ recipients through Day 200 for high‐risk recipients	Conditional (SOT); Strong (HSCT)
Prevention: CMV‐CMI testing	Strong recommendation for R+ kidney recipients to personalize prophylaxis duration	Case‐by‐case determination; potential role in letermovir recipients	Strong (SOT); Conditional (HSCT)
Treatment	Valganciclovir/ganciclovir remains first‐line; thresholds for treatment duration are challenging in the era of sensitive testing	Ganciclovir/valganciclovir is first‐line for treatment; preemptive therapy with weekly monitoring	Strong (both)
Resistant/refractory CMV	Maribavir is the principal alternative for resistance, unless high VLs (prefer foscarnet)	Maribavir is effective with lower toxicity than ganciclovir/foscarnet; second‐line approved for ≥ 12 years	Strong (both)
Resistance testing	Strong recommendation for genotypic testing after ≥ 4 weeks’ exposure and suboptimal response to correctly dosed treatment	Genotypic testing is recommended when resistant or refractory CMV is clinically suspected	Strong (both)
CMVIG	Conditional recommendation for severe/refractory disease, hypogammaglobulinemia, and thoracic transplants	Adjunctive therapy in select scenarios; limited evidence	Conditional (both)
Pediatrics	Prevention strategies include prophylaxis, PET, or surveillance after short‐term prophylaxis; more data are needed on new drugs	Letermovir approved for pediatric patients; drug‐ and weight‐based dosing criteria apply	Strong for available data

Abbreviations: ASTCT, American Society for Transplantation and Cellular Therapy; CMI, cell‐mediated immunity; CMV, cytomegalovirus; CMVIG, cytomegalovirus immunoglobulin; CMV‐QNAT, cytomegalovirus quantitative nucleic acid testing; D+/R−, donor seropositive/recipient seronegative; ECIL, European Conference on Infections in Leukaemia; ESCMID, European Society of Clinical Microbiology and Infectious Diseases; GRADE, Grading of Recommendations Assessment, Development and Evaluation; HSCT, hematopoietic stem cell transplantation; PCR, polymerase chain reaction; PET, preemptive therapy; R+, recipient seropositive; SOT, solid organ transplantation; VL, viral load; WHO, World Health Organization.

^a^ Recommendation strengths per GRADE (SOT) and ESCMID (ECIL) methodologies.

Despite these advances, the “troll of transplantation” [4] remains formidable as late‐onset disease continues to affect high‐risk patients, treatment‐resistant virus remains a threat, and the high costs of both new technologies and personalized treatment approaches can create barriers to optimal care [5, 6].

To discuss these recent advances and ongoing challenges, more than 200 clinicians and researchers from transplant centers and research laboratories around the globe convened at the third International CMV Symposium in Königstein, Germany, on September 12–13, 2025. The 2‐day program featured invited faculty presentations, challenging case discussions with expert audience participation, and abstract presentations from young investigators, fostering cross‐disciplinary dialogue in an intimate setting. Top experts in CMV and transplantation shared their expertise to address the central question of how to apply the updated guidelines across diverse healthcare settings to improve patient outcomes. A particular strength of the symposium was the integration of SOT and HSCT expertise, providing opportunities to translate successful strategies across transplant disciplines while acknowledging population‐specific considerations.

This article synthesizes key discussions from the symposium, with particular focus on areas where evidence‐based recommendations encounter real‐world implementation challenges (Table 2). We examine how recent advances translate to clinical practice, explore barriers to guideline adherence, and identify research priorities to address current gaps in CMV management.

**TABLE 2:** Potential implementation barriers for recommendations in 2025 guidelines.

**Guideline recommendation**	**Primary implementation challenge**	**Real‐world impact**
Preemptive therapy in intermediate‐risk SOT	Weekly CMV monitoring for 12–16 weeks is required; lack of standardized/well‐defined viral load thresholds	Infrastructure burden for laboratories; logistical challenges; institutional heterogeneity in thresholds for therapy initiation
TDM for ganciclovir/valganciclovir	Limited laboratory availability; prolonged turnaround times	Dosing adjustments made empirically without drug level guidance; standard dosing is frequently suboptimal
CMI testing to guide prophylaxis duration	Test availability varies by region; reimbursement is inconsistent	Cost‐effectiveness needs to be demonstrated; access is limited, and a dedicated laboratory capacity is required
Extended letermovir prophylaxis in high‐risk HSCT	High drug acquisition costs; unclear optimal duration	Financial burden limits adoption; practice variation in duration based on institutional resources
Surveillance after prophylaxis in patients with high‐risk SOT	Weekly monitoring for 8–12 weeks post‐prophylaxis	Logistical challenges; late‐onset disease still occurs despite surveillance
Genotypic resistance testing	Prolonged turnaround times in some labs; requires adequate viral load for sequencing	Treatment decisions are often needed before results are available; limited laboratory availability
Letermovir prophylaxis in D+/R− SOT	High drug costs; limited data in non‐kidney organs and R+ recipients	Investigational status for non‐kidney organs despite theoretical advantages; cost‐benefit analyses needed

Abbreviations: CMI, cell‐mediated immunity; CMV, cytomegalovirus; D+/R−, donor seropositive/recipient seronegative; HSCT, hematopoietic stem cell transplantation; R+, recipient seropositive; SOT, solid organ transplantation; TDM, therapeutic drug monitoring.

## 2 CMV Prevention Strategies: Evolution and Implementation

### 2.1 SOT: Balancing Efficacy and Practicality

In the opening session, Camille N. Kotton reviewed the 4th International Consensus Guidelines for SOT, updated for the first time since 2018 [1]. The fundamental approach to prevention remains risk‐stratified based on donor (D) and recipient (R) CMV serostatus; new data and treatment options have refined the choice between prophylaxis and preemptive therapy. For high‐risk heart and lung transplant recipients (seropositive donors to seronegative recipients; D+/R−), universal prophylaxis with valganciclovir remains the recommended approach, whereas for kidney and liver recipients, prophylaxis or preemptive therapy are considered equally effective [1]. Duration of prophylaxis varies by organ, typically ranging from 3 to 6 months for kidney and liver recipients to 12 months for lung recipients, reflecting greater CMV disease risk and immunosuppression required in thoracic organ transplantation. For intermediate‐risk recipients (R+), the guidelines acknowledge that prophylaxis or preemptive therapy could be acceptable strategies depending on patient‐specific factors, logistics, and institutional standards. Elisa Ruiz‐Arabi reviewed the debate between the two approaches, which remains ongoing.

Late‐onset CMV disease remains a persistent challenge even with extended prophylaxis, as demonstrated in Canadian data presented in Ruiz‐Arabi's talk, showing CMV infection developed in 34% of D+/R− kidney transplant recipients despite a median of 180 days of valganciclovir prophylaxis [7]. These findings may reflect maintenance immunosuppression and myelotoxicity. Letermovir offers an alternative with a distinct mechanism of action and more favorable myelotoxicity profile, and it demonstrated noninferiority to valganciclovir in preventing CMV disease in D+/R− kidney transplant recipients (10.4% vs. 11.8%, respectively) in the recent multicenter pivotal trial [8]. The introduction of letermovir as a prophylactic agent represents a notable advance in the field, although its role in SOT remains restricted to the D+/R− kidney transplant population. Small studies in heart and lung transplant recipients have suggested potential efficacy [9, 10, 11, 12], but further study is needed to assess breakthrough infection and late‐onset disease risk. Kotton also noted letermovir does not provide concurrent prophylaxis against other herpesviruses, requiring supplementation with acyclovir or valacyclovir. Furthermore, despite cost‐effectiveness analyses showing improved survival, reduced hospitalizations, and favorable total cost of care [13, 14, 15, 16], letermovir acquisition costs remain prohibitive compared with generic valganciclovir in many settings.

Preemptive therapy provides an advantage of avoiding the toxicity risk associated with prolonged exposure to antivirals, but at the cost of significant logistical challenges. For example, it requires frequent visits, and the 12–16 weeks of CMV DNAemia monitoring required for effective preemptive therapy create resource burdens for both patients and healthcare systems (e.g., laboratory infrastructure and associated costs). Ruiz‐Arabi also noted the lack of universal consensus on when to initiate preemptive therapy, noting heterogeneity in preferred or available testing approaches and variable viral load criteria for initiating preemptive therapy across institutions. The SOT guidelines recommend that individual centers establish their own thresholds based on D/R status and assay characteristics [1]. As another option, a “hybrid” approach is emerging that combines initial prophylaxis with subsequent intensive monitoring in higher‐risk recipients. Although this strategy may offer practical advantages in resource‐limited settings, supporting data remain limited.

Pediatric transplant recipients present unique challenges for CMV prevention, and the updated SOT guidelines recommend CMV serology be performed in all children [1]. Interpretation in infants requires careful consideration, and CMV seropositivity in this age group should be considered highest‐risk categorization given the possibility that passively acquired maternal antibodies may obscure true CMV‐naive status. Prevention strategies in this cohort mirror adult approaches, with adjustments for developmental pharmacology, higher viral loads, and prolonged time to viral clearance observed in pediatric populations.

### 2.2 HSCT: Prophylaxis in the Letermovir Era

In contrast with its more limited status in SOT, letermovir has fundamentally transformed CMV prevention in allogeneic HSCT and is considered standard prophylaxis for R+ by both ECIL and ASTCT guidelines [2, 3]. In the pivotal Phase 3 trial, letermovir led to a 44% reduction in clinically significant CMV infection through 24 weeks posttransplant [17], and remarkably demonstrated profound positive impacts on mortality and other transplant‐related outcomes in a subsequent real‐world analysis. Jan Styczynski presented a meta‐analysis by Roy Chemaly's group demonstrating significant reductions in all‐cause mortality (27%), non‐relapse mortality (35%), graft‐versus‐host disease (GVHD; 48%), and CMV‐related hospitalizations (94%) [18]. The data suggest letermovir effectively abrogates the impact of recipient CMV seropositivity to approximate that of seronegative recipients.

However, Daniel Teschner noted that the Phase 3 trial also revealed a concerning pattern in which some patients experienced CMV reactivation after standard 100‐day prophylaxis [17]. Extending prophylaxis to 200 days was subsequently shown to provide excellent suppression, yet some patients still experienced reactivation after discontinuation [19]. These findings prompted faculty discussion regarding the optimal duration of prophylaxis and whether extended suppression simply delays rather than prevents CMV reactivation. The discussion revealed that letermovir prophylaxis generally varies by center, with longer durations (up to 200 days) typically reserved for patients with persistent high‐risk features such as ongoing immunosuppression for GVHD, mismatched or haploidentical transplant, absence of CMV‐specific CMI, or prior CMV disease.

Other important clinical considerations for letermovir prophylaxis based on its unique pharmacologic properties as a viral terminase inhibitor and interaction profile were presented. One consideration is the presence of transient low‐level CMV DNAemia (“blips,” typically ≤ 1500 IU/mL), representing abortive viral replication during letermovir prophylaxis [20], which does not indicate resistance or prompt preemptive therapy [2]. Resistance during primary letermovir prophylaxis is uncommon (incidence < 2% in Phase 3 trials) [21], but can develop if subtherapeutic levels are provided, such as with treatment interruption or malabsorption [1]. Letermovir is also subject to drug‐drug interactions as it can increase exposures to cyclosporine, tacrolimus, and sirolimus [22]. The cyclosporine interaction is bidirectional and requires a 50% letermovir dose reduction with coadministration [1, 23]. Finally, the high specificity of letermovir for CMV has resulted in acyclovir or valacyclovir supplementation becoming standard practice in HSCT centers to prevent other herpes simplex and/or varicella‐zoster virus infections.

Letermovir is not currently recommended for R− HSCT recipients regardless of donor serostatus due to low CMV reactivation risk and lack of controlled clinical data. For R− recipients, alternative prophylactic agents such as high‐dose acyclovir or valacyclovir may be considered, although data supporting their use for CMV prevention are limited [1].

An emerging practice highlighted in the 2025 ECIL guidelines is pre‐HSCT polymerase chain reaction (PCR) screening, as pretransplant CMV DNAemia has been identified as a risk factor for posttransplant CMV events, affecting approximately 11% of R+ patients, with lymphopenia prior to transplant being a key predisposing factor [2]. The guidelines recommend pretransplant PCR testing to inform risk stratification and management decisions, although optimal strategies for managing patients with detectable viremia remain under investigation.

Evidence is also accumulating supporting the use of letermovir in the pediatric HSCT population, although these patients have some distinct management challenges, and dosing parameters have yet to be defined. One consideration is the unique CMV epidemiology among pediatric patients undergoing HSCT. Styczynski presented data from 13 international studies representing 4600 pediatric HSCT recipients, revealing that 37.8% are R− compared to 22.6% of adult recipients; thus, a substantial proportion fall outside letermovir indications for R+ patients. Children also demonstrate age‐dependent T‐cell immunity against CMV, with lower baseline immunity than adults even when R+ [24, 25], which may affect decisions regarding prophylaxis duration and follow‐up monitoring.

The 2025 ECIL guidelines also address CMV management in recipients of chimeric antigen receptor T‐cell therapy (CAR‐T), an expanding population with CMV risk profiles lower than HSCT, yet still significant due to lymphodepleting conditioning [2]. Eleftheria Kampouri reviewed emerging data showing CMV reactivation is frequent in CAR‐T recipients, particularly patients receiving corticosteroids or BCMA‐targeted products, although end‐organ disease remains uncommon [26]. Current recommendations focus on monitoring seropositive patients, with active surveillance recommended between Weeks 2 and 6 post‐infusion for high‐risk individuals [2]. Optimal prevention strategies remain undefined, representing an area of active investigation.

### 2.3 Bridging Evidence to Practice: Persistent Challenges Remain

Translating updated guidelines into clinical practice emerged as a central theme throughout the symposium. Faculty discussions highlighted persistent challenges in several key areas, including resource accessibility (Table 2) and heterogeneity among patient populations and healthcare systems that can limit the implementation of evidence‐based protocols. These challenges are particularly evident with preemptive therapy, which, despite the advantages of reduced drug toxicity and treatment costs, requires testing infrastructure that may be unavailable in some regions. Similarly, therapeutic drug monitoring (TDM), which is recommended during ganciclovir and valganciclovir prophylaxis in populations susceptible to variable drug exposure with standard dosing (i.e., pediatric patients, or those with reduced kidney function/dialysis or cystic fibrosis) [1], also requires a robust infrastructure. In the absence of TDM, clinicians must adjust dosing empirically, which carries the risk of subtherapeutic or toxic exposures.

During the symposium, faculty presented patient cases highlighting some potential effects when guideline‐recommended approaches are not implemented (Table 3). Nicolas Mueller presented a 38‐year‐old heart recipient who had persistent low‐level CMV DNAemia during valganciclovir prophylaxis and developed resistance; the challenges of interpreting and acting on low‐level CMV DNAemia were evident when CMV retinitis developed, highlighting the importance of an interdisciplinary approach. Alaa Atamna presented a 51‐year‐old kidney recipient with breakthrough viremia in whom sequential resistance to ganciclovir then maribavir developed, likely due to the institutional low‐dose prophylaxis protocol that deviated from risk‐stratified guideline recommendations.

**TABLE 3:** Selected cases illustrating complexities of translating CMV guidelines to clinical practice^a^.

**Patient (Presenter)**	**Clinical scenario/ key challenge**	**Treatment sequence/ management approach**	**Key learnings**	**Guidelines‐to‐practice considerations**
38‐year‐old male, heart transplant; D+/R− (Mueller)	Persistent low‐level viremia during prophylaxis progressing to CMV retinitis; resistance detected late, challenge of interpreting and acting on low‐level DNAemia; patient followed in different institutions	VGCV was prescribed by the primary transplant team at a prophylactic dose level; resistance testing was performed, but the result was initially missed	Optimal care for persistent/complicated CMV includes multidisciplinary care with consultation by infectious disease specialists to ensure appropriate treatment and timely response to changes	Illustrates the importance of having effective institutional systems/protocols and care coordination to adhere to guideline‐recommended treatment; continuous care across different institutions
51‐year‐old male, kidney transplant; D+/R− (Atamna)	Breakthrough viremia during low‐dose prophylaxis (center protocol); sequential development of GCV then MBV resistance	GCV (for breakthrough infection) → FOS → MBV (resistance at Week 6 after initial response) → FOS + LET (secondary prophylaxis) → CMVIG added for persistent low‐level viremia; resolution after CMVIG	MBV resistance can emerge rapidly, particularly with high viral loads; close virologic monitoring during MBV therapy is essential; CMVIG may provide benefit as adjunctive therapy	Illustrates deviation from guideline‐recommended, risk‐stratified dosing due to institutional protocols likely contributing to breakthrough, and access barriers to newer, guideline‐recommended agents, delaying optimal therapy
57‐year‐old male, lung transplant for IPF; D+/R− (Pascale)	Preemptive monitoring strategy selected over guideline‐recommended prophylaxis due to renal impairment; subsequent CMV pneumonia with compartmentalized resistance (BAL positive, blood negative)	VGCV (initial episode); leukopenia prompted prophylaxis cessation; GCV + CMVIG → FOS + CMVIG for pneumonia after BAL resistance detected	Resistance testing should include BAL when pulmonary involvement is suspected; compartmentalized resistance may require site‐specific sampling to detect	Illustrates how comorbidities (renal impairment, leukopenia) may preclude guideline‐recommended prophylaxis, and how approved indications for newer agents (e.g., letermovir) may lag behind clinical need
28‐year‐old male, heart transplant for Kearns‐Sayre syndrome; D+/R− (Just‐Lauer)	High‐level viremia (> 450 000 copies/mL) with neurological symptoms; underlying mitochondrial disorder raised concerns about antiviral toxicity; concurrent DSA development	GCV with CMVIG (selected as lowest‐risk option); MMF cessation, IVIG for DSA	CMVIG should be considered when standard antivirals are contraindicated	Illustrates how certain underlying conditions may pose disease‐specific safety concerns, precluding safe administration of guideline‐recommended therapy
29‐year‐old female, allo‐HSCT for high‐risk AML; D+/R+ (Lohmeyer)	CMV reactivation during steroid treatment for GVHD; required differentiation of etiology for hepatitis vs. colitis (GVHD vs. CMV); competing need to reduce immunosuppression for relapse prevention	LET prophylaxis (Day 100) → VGCV + Cytotect (for concurrent GVHD and CMV) → MBV (selected for outpatient management per patient preference); resolution occurred	Overlapping symptoms between CMV and other conditions (e.g., GVHD) can occur, requiring careful differentiation; patient‐specific circumstances and/or preferences (including need for outpatient management) must be considered during treatment decisions	Illustrates how competing clinical priorities (relapse prevention vs. GVHD management vs. CMV control) and patient circumstances/preferences can require individualized decision‐making that balances guideline recommendations against real‐world constraints

Abbreviations: AML, acute myeloid leukemia; BAL, bronchoalveolar lavage; CMV, cytomegalovirus; CMVIG, cytomegalovirus immunoglobulin; D+/R−, donor seropositive/recipient seronegative; DSA, donor‐specific antibodies; FOS, foscarnet; GCV, ganciclovir; GVHD, graft‐versus‐host disease; HSCT, hematopoietic stem cell transplantation; IPF, idiopathic pulmonary fibrosis; IVIG, intravenous immunoglobulin; LET, letermovir; MBV, maribavir; MMF, mycophenolate mofetil; VGCV, valganciclovir.

^a^ See Supporting Information S1 for full case details.

Patient heterogeneity can affect guideline adherence at multiple levels. At the population level, studies suggest geographic variation in CMV seroprevalence, which exceeds 90% in some regions compared with 50%–60% in others [27, 28, 29], fundamentally altering risk‐benefit calculations for prevention strategies. At the patient level, genetics, medical history, and current health status (i.e., comorbidities) must be considered when following guideline‐recommended approaches. Recipients of highly sensitized or human leukocyte antigen (HLA)‐mismatched grafts face disproportionate risk of CMV infection, while patients receiving novel immunosuppressive regimens may have risk profiles inadequately captured by existing literature. Furthermore, pediatric transplant recipients require age‐ and weight‐specific dosing adjustments.

To illustrate how individual patient characteristics may supersede guideline‐recommended therapy, Renato Pascale presented a case of a 57‐year‐old lung recipient with renal impairment who underwent preemptive monitoring after guideline‐recommended prophylaxis resulted in leukopenia (Table 3). This was also evident from a case series by Amina Abu‐Omar in which CMVIG was used in patients with premature discontinuation of valganciclovir prophylaxis due to leukopenia [30]. Additionally, Isabell Just‐Lauer presented a 28‐year‐old heart recipient with high‐level viremia who was given CMVIG instead of additional antivirals that were considered risky due to underlying Kearns‐Sayre syndrome (a rare mitochondrial disorder) (Table 3).

Thus, despite the essential frameworks guidelines provide for broad patient populations, real‐world challenges such as resource availability, institutional policies, and individual patient characteristics must be considered when determining the optimal approach for CMV prevention.

## 3 Managing Resistant and Refractory CMV

Nassim Kamar began the session on refractory/resistant CMV by reviewing the standardized definitions from the CMV Resistance Working Group [31, 32] that were formalized in the 2025 guidelines, providing essential clarity for both clinical practice and research [1, 2]. Drug resistance is defined as viral genetic alterations reducing susceptibility, based on half maximal effective concentration (EC_50_) in culture. Refractory CMV infection is defined clinically based on viral load that persists or increases ≥ 1 log_10_, or progression or lack of improvement in signs and symptoms after ≥ 2 weeks of appropriately dosed antiviral therapy (Table 4). Drug resistance should be suspected in patients with cumulative antiviral exposure > 4 weeks and treatment failure despite ≥ 2 weeks of appropriately dosed therapy [1], although ganciclovir resistance typically requires > 6 weeks of cumulative exposure to develop in the absence of adverse host factors or high viral loads [33].

**TABLE 4:** Key principles for managing resistant/refractory CMV infection [1, 2, 3].

**Decision point**	**Recommendation**
When to suspect resistance	Cumulative antiviral exposure > 4 weeks with ≤ 1 log_10_ decrease in viral load after ≥ 2 weeks of appropriately dosed therapy.
Resistance testing	Genotypic testing recommended when viral load > 1000 IU/mL; include *UL97*, *UL54*, and *UL56* (if prior letermovir exposure).
First‐line for R/R CMV	Maribavir preferred for most patients; foscarnet for high viral loads (≥ 50 000 IU/mL) or CNS/retinal disease.
Monitoring during maribavir	Close virologic surveillance; viral rebound suggests emergent resistance (86% of patients who experience rebound develop mutations).
Adjunctive therapies	Consider CMVIG for severe disease, drug intolerance, or IgG < 400 mg/dL; immunosuppression reduction. CMV‐specific T cells may be considered when available. For multidrug‐resistant infections, consider special access programs or clinical trials for investigational agents.

Abbreviations: CMV, cytomegalovirus; CMVIG, cytomegalovirus immunoglobulin; CNS, central nervous system; IgG, immunoglobulin G; R/R, refractory/resistant.

Genotypic resistance testing is strongly recommended when refractory infection is suspected, and viral loads > 1000 IU/mL are recommended for reliable sequencing [1]. Ganciclovir‐related resistance is assessed by probing for mutations in the viral kinase gene *UL97* and DNA polymerase gene *UL54*, while letermovir resistance can result from mutations in the terminase gene *UL56*, and maribavir resistance from mutations in *UL27*. Next‐generation sequencing provides high sensitivity for detecting resistance variants with low copy numbers with a short turnaround time [34]. Interpretation of resistance mutations has become increasingly sophisticated, with online resources such as the Comprehensive Herpesviruses Antiviral drug Resistance Mutation Database (CHARMD; https://www.unilim.fr/cnr‐herpesvirus/outils/codexmv/database/all_references) available for querying published phenotypes and predicted drug susceptibilities.

### 3.1 Maribavir: Transforming Management of Resistant Disease

Maribavir represents the most significant advance in managing refractory/resistant CMV in decades. In the pivotal, Phase 3 SOLSTICE trial in 352 patients with refractory/resistant CMV infections after SOT or HSCT, maribavir led to 56% virologic clearance at 8 weeks compared with 26% with investigator‐assigned therapy, which was sustained at 16 weeks (41% vs. 22%) [35]. Real‐world effectiveness data have since confirmed that these positive results translate to the clinic [36, 37, 38, 39, 40], and helped establish maribavir as a first‐line option for refractory/resistant CMV [1]. Given the small number of patients with high viral loads in the SOLSTICE trial and the emergence of maribavir‐resistant virus, guidelines recommend foscarnet for viral loads ≥ 50 000 IU/mL (4.7 log_10_) [1]. Nathan Kasriel presented recent real‐world data from a multicenter cohort of kidney transplant recipients in France that showed high baseline viral loads (> 10 000 IU/mL) and symptomatic infection were associated with poorer outcomes and increased development of resistance, supporting initial induction with foscarnet and transition to maribavir once viral load decreases [41].

Maribavir has a favorable safety profile, including lower overall mortality after 1 year compared with conventional therapies [42]. The most common adverse effect is dysgeusia, which affects about half of the patients who receive the drug but rarely leads to treatment discontinuation [35, 43, 44]. Decreases in glomerular filtration rate have been observed and require monitoring, but are reversible and far less severe than the nephrotoxicity associated with cidofovir or foscarnet [45, 46]. Maribavir is also a moderate cytochrome P450 2C9 (CYP2C9) inhibitor and a substrate of CYP3A; however, aside from antagonistic drug–drug interactions with ganciclovir, other interactions (e.g., tacrolimus) are considered manageable [47, 48]. Maribavir is available as an oral formulation more amenable to outpatient management compared with intravenous (IV) agents. This advantage was nicely illustrated in a case presented by Juliane Lohmeyer of an HSCT recipient with refractory CMV whose circumstances as a young mother favored outpatient therapy, and for whom maribavir resulted in undetectable viral load within 10 days (Table 3).

Despite these benefits, maribavir resistance has emerged as a critical concern. Recent data by Chou et al. showed that 26% of patients develop resistance during treatment, predominantly involving T409M, H411Y, and C480F substitutions in *UL97* [49, 50]. T409M confers an 80‐fold increase in EC_50_ while C480F also confers low‐level cross‐resistance to ganciclovir. Resistance to maribavir occurs more rapidly and frequently than to ganciclovir. Viral rebound during maribavir therapy should immediately raise suspicion of potential resistance, given that 86% of patients in the Phase 3 trial who rebounded developed resistance mutations [49]. This high emergence of resistance, which was illustrated in the case presented by Alaa Atamna (Table 3) highlights the importance of close virologic monitoring during maribavir treatment.

### 3.2 Alternative and Adjunctive Approaches

For infections resistant to multiple antivirals or if maribavir is not available, foscarnet and cidofovir remain salvage options despite significant toxicities. Foscarnet requires close monitoring due to the substantial risk of nephrotoxicity and electrolyte disturbances, while cidofovir requires concomitant probenecid, aggressive hydration, and vigilance for dose‐limiting nephrotoxicity [51].

While the updated SOT guidelines state that CMVIG may be considered for prophylaxis in specific circumstances, such as thoracic organ transplantation, D+/R− serostatus, or severe hypogammaglobulinemia, it can also be used as adjunctive therapy, particularly for severe or life‐threatening disease refractory to antiviral therapy alone. The updated SOT guidelines provide conditional recommendations to use CMVIG in combination with antivirals for resistant disease in patients with hypogammaglobulinemia (immunoglobulin G [IgG] < 400 mg/dL), and as a consideration in thoracic organ transplant recipients with severe disease [1]. Javier Carbone reviewed the immunologic rationale, noting that beyond simple antibody replacement, in vitro data suggest CMVIG may enhance CMV‐specific T‐cell responses through cross‐presentation mechanisms and augment natural killer cell polyfunctional activity [52]. Additional in vitro evidence presented by Amina Abu‐Omar suggests CMVIG facilitates expansion of CMV‐specific T cells [53]. Although randomized clinical trial data remain limited, these observations provide a theoretical basis for CMVIG use in patients with impaired cellular immunity and were illustrated in the case presented by Isabell Just‐Lauer. This was a 28‐year‐old male heart transplant recipient with Kearns‐Sayre syndrome in whom high‐level CMV viremia with neurological manifestations developed despite prophylaxis (Table 3). Management required balancing CMV control against rejection risk during the emergence of donor‐specific antibodies. The team elected to add CMVIG to ganciclovir therapy because its favorable safety profile was considered advantageous for a patient with underlying mitochondrial disease, which raised concerns about metabolic toxicity from other options. Viral loads declined and neurological symptoms resolved, although the patient subsequently required treatment for rejection after mycophenolate mofetil was held. Nassim Kamar also contributed real‐world experience by describing how his center incorporates CMVIG into individualized management for thoracic transplant recipients and patients with refractory/resistant infection, which has provided therapeutic responses even in challenging cases when used according to previously described protocols [54].

## 4 Precision Medicine: CMI Testing

### 4.1 The “Delicate Dance” of Immunosuppression and CMV

A symposium session on the current state of immunoguided therapy began with Brad Gardiner introducing immunoguided management as a delicate dance requiring precise timing, coordination, and balance to maintain a level of immunosuppression sufficient to prevent transplant rejection while also controlling viral replication. He emphasized during his talk that, despite decades of experience and sophisticated prevention strategies, clinicians often remain in the dark when attempting to predict which patients will experience clinically significant CMV reactivation. A key insight highlighted during the presentation is how the “net state of immunosuppression” proves more important than the specific immunosuppressive agents used when determining CMV risk. This cumulative effect encompasses not only maintenance immunosuppression but also treatment for rejection, concurrent infections, comorbidities, and evolving immune reconstitution in HSCT recipients. The dynamic nature of the process requires constant adjustment rather than fixed protocols, whereas traditional approaches rely on static risk stratification based on donor‐recipient serostatus and organ type. This gap between the dynamic reality of immune function and static prevention strategies has driven interest in biomarkers that can personalize CMV management.

### 4.2 CMI Testing in SOT: From Research to Guidelines

CMV‐specific CMI testing represents one of the most significant advances in precision medicine in the transplant field. Multiple assay platforms are now commercially available to measure T‐cell responses to CMV antigens through different methodologies. QuantiFERON‐CMV is an ELISA‐based platform measuring interferon‐gamma production primarily from CD8+ T cells in response to CMV peptides. ELISpot‐based assays (T‐SPOT.CMV, T‐Track CMV) count individual spot‐forming cells and incorporate both CD4+ and CD8+ T‐cell responses. Flow cytometric assays also detect CMV‐specific CD4+ and CD8+ T cells, with the Viracor CMV assay being the first FDA‐approved CMI assay. Despite methodological differences, all platforms demonstrate negative predictive value exceeding 90%, suggesting that R+ recipients with reactive CMV‐CMI tests face a low risk of clinically significant CMV infection.

In her presentation on immunodiagnostics, Martina Sester highlighted how the 2025 SOT guidelines provide the first strong recommendation for use of CMV‐CMI testing in clinical practice, which is recommended for guiding the duration of prophylaxis R+ kidney recipients [1]. This recommendation is supported by evidence from multiple randomized controlled trials demonstrating that patients with reactive CMV‐CMI can safely discontinue prophylaxis earlier than with standard protocols, allowing for antiviral exposure to be reduced by approximately 30–50 days while maintaining protection against CMV disease [55, 56, 57].

Clinical implementation of CMV‐CMI follows a straightforward algorithm in which the R+ kidney recipient is tested during or at the end of standard prophylaxis, with reactive results indicating the patient can discontinue prophylaxis (potentially with continued virologic monitoring), while nonreactive results suggest continuing prophylaxis or transitioning to enhanced surveillance. While test expenses may be offset by reductions in costs for antiviral drug acquisition, toxicity management, and monitoring, formal cost‐effectiveness analyses are needed. The guidelines recommend against using CMI for guiding prophylaxis duration in D+/R− patients. This recommendation is supported by data from the Swiss Transplant Cohort Study, which revealed only 23% of D+/R− kidney patients demonstrated detectable CMV‐CMI at 5 months posttransplant [57], indicating that most patients will require the anticipated duration of prophylaxis.

Other indications for using CMV‐CMI include assigning CMV infection status in individuals with potential passive immunity [58] or early identification of R+ patients at low risk for subsequent reactivation episodes [59]. Observations from case series and small interventional trials suggest CMV‐CMI may also be used to guide treatment duration and predict relapse after treatment discontinuation, although further studies are needed to better understand its role in this setting. Limitations of CMV‐CMI include minimal evidence supporting its use in organs other than kidney, and responses fluctuate with changes in immunosuppression intensity, particularly during rejection treatment or concurrent infections, requiring careful timing considerations when interpreting results [1].

### 4.3 CMI Testing in Cellular Therapy Recipients: Navigating Unique Complexities

Roy Chemaly presented the current status of CMV‐CMI testing in HSCT populations, which remains more complex and less defined than in SOT. Unique challenges for CMV‐CMI testing in this setting include dynamic immune reconstitution, profound immunodepletion from conditioning regimens, and ongoing risk of GVHD. Both 2025 ECIL and ASTCT guidelines acknowledge the potential value of CMV‐CMI monitoring in HSCT, yet both stop short of providing strong recommendations, instead suggesting that the decision be made on a case‐by‐case basis [2, 3].

CMV‐CMI testing during letermovir prophylaxis also raises unique considerations. While letermovir effectively prevents CMV reactivation, it may delay development of CMV‐specific T‐cell immunity, possibly related to decreased CMV antigen exposure [60]. Optimal timing for CMV‐CMI testing relative to letermovir discontinuation remains undefined, although a recent analysis from the pivotal letermovir clinical trial provided initial insights. Results showed that CMI positivity rates were lower at 200 days post‐HSCT with letermovir than placebo (48.8% vs. 74.5%), and rates converged by Week 48, suggesting immune recovery occurs after prophylaxis completion. However, the predictive utility of the assay appeared limited in this setting [61].

Beyond traditional HSCT, CMV‐CMI testing may also have utility in recipients of CAR‐T, a population now addressed in the 2025 ECIL guidelines due to similar immunosuppression‐related CMV risk [2]. Eleftheria Kampouri reviewed data demonstrating that low CMV‐specific T‐cell responses at Week 2 post‐infusion identify patients at higher reactivation risk, suggesting CMV‐CMI assays could help stratify this expanding population [26].

### 4.4 Beyond CMV‐Specific Immunity: Global Immune Monitoring

Beyond CMV‐specific responses, biomarkers of global immune function may further refine risk stratification [62]. Brad Gardiner presented data on the absolute lymphocyte count (ALC) and mitogen response included in QuantiFERON‐CMV testing, which assesses overall T‐cell function independent of CMV specificity. Patients with profoundly diminished mitogen responses demonstrated markedly higher CMV disease risk and 6‐fold increased risk for serious opportunistic infections [63, 64]. Notably, this information is automatically generated with every QuantiFERON‐CMV test, providing additional clinical insights without requiring separate testing. Low ALC has been associated with CMV risk in a growing number of studies, and this widely available biomarker has good potential for predicting CMV risk and individualizing prevention strategies [65, 66].

Hypogammaglobulinemia, affecting 15%–45% of transplant recipients depending on organ and immunosuppression regimen, represents another dimension of immune dysfunction [67]. The 2025 guidelines recommend IgG measurement in high‐risk SOT recipients within the first month posttransplant, with retesting when CMV proves difficult to control [1], as severe hypogammaglobulinemia (IgG < 400 mg/dL) is associated with increased CMV disease risk. Javier Carbone emphasized that this provides rationale for considering CMVIG or intravenous immunoglobulin (IVIG) replacement in affected patients, particularly thoracic transplant recipients or patients with refractory/resistant infection.

Torque teno virus (TTV) load was also highlighted as an emerging biomarker, as viral burden corresponds with immunosuppression intensity. A systematic review of 23 studies demonstrated that high TTV loads were associated with increased infection risk, including CMV, while low TTV loads correlated with rejection risk, suggesting utility in guiding immunosuppression adjustments [68]. However, standardized cutoffs and clinical algorithms remain under development [69, 70, 71].

### 4.5 Implementation Barriers and the Path Forward

The infrastructure and resource barriers described earlier for preemptive monitoring apply equally to CMV‐CMI testing. Availability varies dramatically by region, with many centers lacking access to commercial platforms, and specialized immunology testing may require different laboratory infrastructure than standard assays. Cost and reimbursement present additional obstacles, with cost‐effectiveness yet to be established and insurance coverage inconsistent between regions. Further standardization of optimal timing, frequency, and clinical algorithms may facilitate broader adoption.

Symposium panels emphasized that successful precision medicine in CMV management will require integrating multiple biomarkers rather than relying on any single test. CMV‐specific immunity, global immune function, clinical risk factors, and the evolving posttransplant course must all inform individualized decisions. As the field moves from fixed‐duration prophylaxis toward personalized strategies, the “delicate dance” Gardiner described becomes increasingly sophisticated, demanding continuous assessment and adjustment.

## 5 Adoptive Cellular Therapies: From Salvage to Standard

In a session on adoptive cellular therapies, Hannah Kaminski provocatively polled the audience on whether CMV should be viewed primarily as a viral infection or an immunological disorder. The response was divided, reflecting an evolving perspective: while antiviral agents suppress viral replication, optimal long‐term control may fundamentally depend on reconstituting effective CMV‐specific immunity. This concept provides the rationale for adoptive cellular therapies, which aim to restore the immune response rather than simply suppress the virus.

### 5.1 Virus‐Specific T‐Cell Therapy: Maturation to Broad Accessibility

Virus‐specific T‐cell (VST) therapy has evolved substantially over the last decade, using ex vivo isolation or antigen stimulation to generate polyclonal T‐cell populations with single or multiple viral specificities capable of recognizing and eliminating CMV‐infected cells. Patrizia Comoli discussed the recent progression from labor‐intensive research protocols to increasingly accessible clinical products.

In HSCT recipients, VST therapy has demonstrated response rates approaching 70% in patients with refractory/resistant CMV infection, with efficacy shown for both donor‐derived and third‐party products [72]. The 2025 HSCT guidelines provide a conditional recommendation for VST therapy in this setting, grading evidence as weak but acknowledging potential clinical utility [2, 3]. For SOT recipients, where donor‐derived T cells are available only in living donor settings, Phase 2 trial data revealed 65% overall response, with organ‐specific variations in complete response rates for kidney (40%), liver (66%), and heart and lung (50%) [73].

Third‐party VST banks represent a transformative advance, with cryopreserved products from HLA‐matched seropositive donors enabling rapid deployment. Recent trials in HSCT comparing donor‐derived versus third‐party VSTs showed equivalent response rates of 56%, validating the off‐the‐shelf approach [74]. Theoretical GVHD concerns have not materialized clinically, with acute rejection in only 3% of SOT recipients [73].

T‐cell therapy manufacturing protocols continue to evolve toward greater efficiency. Kaminski reported preliminary data showing that traditional 2‐ to 8‐week expansion protocols can be accelerated to days using magnetic bead or multimer‐based selection methods, with comparable efficacy.

Critical questions remain, including whether VSTs should follow antiviral failure or be used earlier in high‐risk patients, and what optimal dosing should be if the initial response is inadequate. Faculty discussions emphasized the value of CMV‐CMI monitoring to guide repeat dosing, as rising CMV‐specific immunity indicates successful engraftment, while persistently absent responses may warrant dose escalation or additional infusions. The need for predictive biomarkers to identify which patients will benefit most remains an active area of investigation.

Both SOT and HSCT guidelines provide weak recommendations for VST therapy due to limited data, restricted availability, and lack of prospective randomized trials comparing it against standard therapies [1, 2]. Yet consistent efficacy in patients who have exhausted conventional options, combined with favorable safety profiles, supports continued development and expanded access.

### 5.2 Gamma‐Delta T Cells: Universal Donor Potential

Kaminski also introduced gamma‐delta (γδ) T cells as a potentially transformative approach addressing key limitations of conventional VST therapy. These unconventional T cells recognize CMV‐infected cells through major histocompatibility complex‐independent mechanisms, meaning they do not require HLA matching between donor and recipient. This universal donor potential could enable true off‐the‐shelf products without the matching requirements that currently limit VST accessibility.

Kaminski's group has developed protocols to expand γδ T cells from healthy donors, with preclinical data demonstrating anti‐CMV activity and plans for early‐phase clinical trials in transplant recipients. If successful, this platform could allow a single donor to provide therapy for multiple recipients with superior manufacturing scalability than current VST approaches. However, fundamental questions remain about which mechanisms underlie the specific expansion of these cells during CMV infection.

### 5.3 Integration With Antiviral Therapy and Future Directions

Cellular therapies represent an emerging option for patients with refractory/resistant CMV infection, with faculty discussing potential combination strategies with antiviral agents. The ability to reduce immunosuppression, which is often not possible in SOT recipients with marginal graft function or HSCT recipients with active GVHD, can be partially compensated for by infusing virus‐specific immunity. As previously noted, CMVIG may enhance cellular therapy efficacy through improved antigen presentation or T‐cell activation, although prospective data remain limited.

The 2025 guidelines acknowledge the potential of cellular therapy while also noting substantial barriers to widespread implementation [1, 2, 3]. Regulatory pathways vary dramatically by region, manufacturing costs reach tens of thousands of dollars per patient, and reimbursement models remain undefined. Infrastructure requirements for specialized cell therapy manufacturing further limit accessibility.

Nevertheless, the trajectory is clear: cellular therapies are transitioning from last‐resort salvage to legitimate treatment options in the CMV armamentarium. The field is moving beyond simply asking whether these therapies work to addressing how, when, and in whom they should be deployed to maximize benefit while managing cost and complexity.

## 6 CMV Vaccines: Renewed Momentum After Decades of Setbacks

After more than 60 years of research, CMV vaccine development has reached an inflection point with multiple platforms in late‐stage trials [75]. Matt Reeves provided an update on the current landscape, noting that genuine optimism now exists that effective vaccines may finally reach clinical practice within the coming decade.

### 6.1 Vaccine Platforms Under Investigation

#### 6.1.1 mRNA‐Based Vaccines

The most advanced candidate is Moderna's mRNA‐1647, encoding glycoprotein B (gB) and the pentamer complex (gH/gL/*UL128‐131*) involved in viral entry [76]. The pentamer enables broad cell tropism, while gB serves as the fusion protein essential for infection of all cell types. In early studies, mRNA‐1647 induced both T‐cell and B‐cell responses in healthy adults, with stronger neutralizing antibody responses compared with previous gB/MF59 formulations [77]. However, gB‐specific antibodies generated by mRNA‐1647 did not include the same correlates of protection identified in earlier gB/MF59 trials, and whether alternative immune responses will prove protective remains to be determined. In late 2025, the Phase 3 CMVictory trial (NCT05085366) investigating the prevention of congenital CMV in women of childbearing age was stopped due to a lack of efficacy after enrolling more than 7400 women. Results from the ongoing Phase 2 trial in HSCT recipients are expected soon [1, 3].

#### 6.1.2 Modified Vaccinia Ankara Platform

The Triplex vaccine uses Modified Vaccinia Ankara (MVA) to express three immunodominant CMV antigens: pp65, IE1, and IE2 [78]. Unlike antibody‐focused strategies, this platform aims to generate robust T‐cell responses. A Phase 2 trial in CMV‐seropositive HSCT recipients demonstrated approximately 50% reduction in CMV events through Day 100 [79]. The most innovative application involves vaccinating HSCT donors prior to transplantation, capitalizing on the established importance of donor immunity in preventing recipient CMV infection in this patient population. This donor vaccination strategy is being expanded to additional settings: the Phase 2 COLT trial (NCT06075745) is currently enrolling CMV‐seronegative liver transplant recipients, measuring reduction in antiviral drug use during the first 100 days posttransplant, with results projected for 2027 [1].

#### 6.1.3 The Protein‐Based Vaccine Legacy

Reeves described how the gB/MF59 adjuvanted‐subunit vaccine achieved approximately 50% efficacy across three clinical trials [80, 81, 82]. Subsequent investigation revealed gB antibody titer as the primary correlate of protection, yet there was no correlation with traditional neutralizing antibody responses, suggesting that alternative immune mechanisms mediate efficacy [83, 84]. Despite proven efficacy, gB/MF59 was not advanced commercially as it failed to achieve sufficient efficacy for licensure. Multiple pharmaceutical companies continue to develop gB‐based candidates, often incorporating additional antigens to boost immunogenicity beyond the 50% plateau.

### 6.2 The Efficacy Paradox

Discussions raised the provocative issue of industry efficacy thresholds versus clinical significance in transplant medicine. Clinicians expressed that 50% efficacy, considered insufficient for industry standards, would nonetheless transform transplant practice by substantially reducing the need for interventional therapy. Reeves referenced the observation by Plotkin et al. that a vaccine protecting a minority of renal transplant patients, or reducing severe disease risk even without preventing infection entirely, still has clinical value [85]. Vaccine development has been driven primarily by the congenital CMV prevention market, in which higher efficacy standards are invoked for preventing maternal–fetal transmission, a consideration largely irrelevant to transplant recipients, yet one that directs financial incentives away from this population. Attendees suggested that separating development pathways and allowing lower efficacy thresholds for transplant indications could accelerate vaccine availability for immunocompromised patients who face the greatest CMV‐related morbidity and mortality.

### 6.3 Implementation Considerations

When vaccines reach the market, key questions will include optimal timing relative to transplant, duration of protection, cost, and integration with existing prevention strategies. Ongoing investigation of the Triplex platform and continued investment in multiple approaches maintain hope that effective vaccines may yet reach clinical practice.

## 7 Conclusion: From Guidelines to Bedside and Beyond

The 2025 International CMV Symposium occurred at a pivotal moment, with three major guidelines published within the year establishing an unprecedented consensus on evidence‐based CMV management. Yet implementation challenges emerged as a recurring theme, as presenters and attendees grappled with translating recommendations into diverse clinical settings in which infrastructure, economics, and institutional capacity vary dramatically.

Significant recent progress is undeniable: letermovir has transformed HSCT outcomes, maribavir provides effective salvage therapy for resistant disease, and CMV‐CMI testing enables personalized management. Prevention has evolved toward precision medicine while cellular therapies and vaccines advance through clinical trials. Yet formidable challenges persist, including late‐onset disease despite extended prophylaxis, increasing resistance risk with expanding antiviral use, and economic barriers creating gaps between evidence and practice globally.

Several research priorities emerged (Table 5). Oriol Manuel highlighted intriguing recent data showing that 20%–50% of D+/R− solid organ transplant recipients never develop detectable CMV infection [86], which may hold clues for future preventive measures. The European HORUS consortium is investigating whether machine learning can identify these “pseudo low‐risk” patients who may not require intensive prophylaxis [87]. Ongoing vaccine and immunoglobulin trials will determine whether prevention can finally move beyond antiviral drugs alone, while integration of multiple immune biomarkers promises more sophisticated risk stratification.

**TABLE 5:** Current unmet needs and research priorities in CMV management.

**Domain**	**Unmet need**
Disease burden	Posttransplant CMV infection persists despite decades of advances.
Guideline implementation	Translation of new (2025) guideline updates into diverse clinical settings is challenged by variable infrastructure, economics, and institutional capacity.
Prevention strategies	Optimal duration of extended prophylaxis is not definitively established; late CMV remains a concern in high‐risk patients. Head‐to‐head comparisons of prophylaxis vs. preemptive therapy approaches are needed. Evidence on letermovir for non‐kidney SOT organs and R+ recipients remains limited.
Antiviral management	Despite improved toxicity, new agents introduce new challenges such as resistance and access/cost (e.g., maribavir resistance emerges more frequently than with ganciclovir). Resistant/refractory CMV infection remains a significant issue for some patients. Standardized viral load thresholds are needed to define when preemptive therapy initiation is needed.
Immune monitoring	Immune monitoring capabilities are limited by a lack of widespread availability and broad expertise, and a lack of a standardized CMI assay.
Vaccination	Lack of effective CMV vaccines remains a major deficit despite recent development efforts.
Translation of clinical evidence	Large, pragmatic studies (“mega‐trials”) are needed to definitively answer key clinical questions with robust sample sizes.

Abbreviations: CMI, cell‐mediated immunity; CMV, cytomegalovirus; R+, recipient seropositive; SOT, solid organ transplantation.

Manuel's closing keynote crystallized a central message with his passionate call for large pragmatic studies (“mega‐trials”) capable of definitively answering key clinical questions. Drawing parallels to cardiology's success with registry‐based trials randomizing hundreds of thousands of patients, he argued that the transplant community must embrace similar collaborative approaches to strengthen the evidence base supporting management decisions.

The integration of SOT and HSCT communities proved particularly valuable, with the success of letermovir in HSCT informing ongoing SOT trials and CMV‐CMI‐guided management experience potentially translating across disciplines.

As the field writes the next chapter in CMV management, the key messages of the symposium were clear: the armamentarium exists, and progress is real, but systematic implementation and collaborative research remain essential to tame the “troll of transplantation” that has been weakened but not yet defeated. Moving forward will require not only continued therapeutic innovation but also dedicated attention to implementation science, economic realities, and global cooperation to ensure that all transplant recipients benefit from these advances.

## Author Contributions

All authors (scientific committee and members of the International CMV Symposium faculty) developed content for presentation during the International CMV Symposium meeting or moderated sessions, where key points reflected in the manuscript were discussed. The scientific committee (Camille N. Kotton, Martina Sester, and Julian Torre‐Cisneros) developed the manuscript based on content and discussion points shared during the meeting. All members of the International CMV Symposium faculty actively reviewed the manuscript content and approved the manuscript prior to submission.

## Funding

This study was supported by Biotest AG.

## Consent

Written informed consent was not obtained, as no patient‐identifiable information is included.

## Conflicts of Interest

In addition to speaking at the Biotest AG‐sponsored International CMV Symposium 2025, the following disclosures/conflicts of interest statements are provided: Camille N. Kotton: Consultant for Abbott Labs, Biotest, Evrys, Hookipa, Merck, Oxford Immunotec, QIAGEN, and Roche Diagnostics. Julián Torre‐Cisneros: Grant support from MSD, Takeda, and Qiagen to IMIBIC; honoraria for lectures from MSD, Takeda, Qiagen, and Biotest; advisory boards for MSD, Takeda, and Grifols. Research supported by CIBER – Consorcio Centro de Investigación Biomédica en Red‐ (CB 2021), Instituto de Salud Carlos III, Ministerio de Ciencia e Innovación, and Unión Europea–NextGenerationEU. Martina Sester: Grant support from Astellas and Biotest to Saarland University outside the submitted work; honoraria for lectures from Biotest, Takeda, Qiagen, and MSD; advisory boards for Moderna, Biotest, MSD, and Takeda. The International CMV Symposium Faculty: Nikolina Bašić‐Jukić: Honoraria from Biotest, Takeda, and Pliva. Javier Carbone: Research and travel grants from Biotest AG. Roy Chemaly: Consultant, speaker, and/or advisor fees from ADMA Biologics, Merck/MSD, Takeda, Shionogi, AiCuris, Astellas, Moderna, Pfizer, Invivyd, Biotest, Gilead, Symbio, AssemblyBio, IntegerBio, Eurofins‐Viracor, and Ansun Pharmaceuticals; institutional research grants from Merck, Karius, AiCuris, Ansun Pharmaceuticals, Symbio, Takeda, OM1, Genentech, and Eurofins‐Viracor. Brad Gardiner: Honoraria from Qiagen, Takeda, and Biotest. Atul Humar: Honoraria/consulting fees from Takeda, Kamada, Synklino, Biotest, and Merck. Isabell Just‐Lauer: Speakers’ honoraria from AstraZeneca, Abbott, Abiomed, and Biotest. Nassim Kamar: Speaker fees and advisory boards for Alexion, Astellas, AstraZeneca, Biotest, BMS, CSL Behring, Chiesi, Eledon, ExeViR, Gilead, Grifols, Hansa, MSD, GlaxoSmithKline, Pierre Fabre, Medison, Neovii, New Bridge, Roche, Sanofi, Sandoz, Synklino, Takeda, and Zydus. Eleftheria Kampouri: Advisory boards and honoraria from MSD; honoraria from Takeda; conference support from Biotest. Nicolas Mueller: Travel grants from Pfizer and Biotest; advisory boards for Pfizer and MSD; honoraria from MSD and Takeda. Genovefa Papanicolaou: Investigator/research funding from MSD, Symbio, and AiCuris; consulting fees from MSD, Regeneron, Takeda, Genentech, and Symbio. Matthew Reeves: Funding from Moderna supporting studies of CMV vaccine development; co‐inventor on an HCMV patent ‘Immunogenic peptide’ WO2022129937A1. Elisa Ruiz‐Arabi: Travel and/or conference support from Takeda Pharmaceuticals; honoraria for lectures, presentations, speakers bureaus, or educational events from Biotest. Jan Styczyński: Lecture fees from AstraZeneca, Biotest, Gilead, MSD, and Sanofi; advisory boards for AstraZeneca and MSD; conference support from AbbVie, AstraZeneca, Biotest, Gilead, MSD, and Roche. Daniel Teschner: Research grants from Gilead; honoraria and consultancies for AbbVie, BioNTech, Daiichi Sankyo, Gilead, iQone, Jazz, Mikrogen, MSD, Noscendo, Octapharma, Pierre Fabre, Pfizer, Tillotts, and Takeda; honoraria from AstraZeneca, Biotest, F2G, Janssen, Novartis, and Sanofi; travel grants from AbbVie, Astellas, Celgene, Daiichi Sankyo, Gilead, Incyte, Jazz, Medac, MSD, Pierre Fabre, Sanofi, Takeda, and Tillotts. Jose Maria Aguado, Rocío Alonso, Alaa Atamna, Udo Boeken, Patrizia Comoli, Lana Desnica, Corrado Girmenia, Peter Jaksch, Hannah Kaminski, Nathan Kasriel, Nithya Krishnan, Juliane Lohmeyer, Oriol Manuel, Julie Messiaen, Amina Abu Omar, Renato Pascale, and Yusri Taha declare no conflicts of interest.

## Supporting information


**Supplementary File 1**: tid70223‐sup‐0001‐SupMat.docx.

**Supplementary File 2**: Visual Abstract.

## Data Availability

Data sharing is not applicable to this article as no new data were created or analyzed in this study.
