# Supplementary material for: The Troll Is Weakened but Not yet Defeated: An Update on Cytomegalovirus Management in Transplantation From the International CMV Symposium 2025
Source: Transpl Infect Dis. 2026 May 5;28(3):e70223. doi: 10.1111/tid.70223 (PMC13262554; doi:10.1111/tid.70223)
Supplement: Supplementary file 1 — tid70223‐sup‐0001‐SupMat.docx. [file TID-28-e70223-s002.docx]

**SUPPORTING INFORMATION**

**Supplement 1. Expanded patient cases from Table 3**

| **CASE 1-1.** Breakthrough viremia and resistance with delayed recognition in heart transplant *(Presented by Nicolas Mueller)* | |
| --- | --- |
| **Patient history** | 38-year-old male with heart transplant (November 2021).  CMV serostatus D+/R-.  Medical history included treated hepatitis C (cleared pre-transplant) and Hodgkin lymphoma (complete remission).  Renal function fluctuating, but eGFR generally >60 mL/min/1.73 m^2^.  CMV prophylaxis: VGCV 450 mg/day (center protocol, which is below guideline-recommended dose of 900 mg/day for eGFR >60 mL/min/1.73 m^2^).  Prophylaxis discontinuation planned at approximately 4 months post-transplant. |
| **Clinical course** | After prophylaxis discontinuation, low-level viremia (507 IU/mL) detected; VGCV restarted at 900 mg/day.  Viral load initially increased then decreased, but fluctuating pattern persisted.  Resistance testing detected *UL97* mutation (K530N) conferring GCV resistance; however, result was not reviewed and no action taken; no immediate consequences as DNAemia was controlled at this time.  Patient changed hospitals but returned in January 2023 with visual symptoms; diagnosed with CMV retinitis of the left eye (viral load 7290 IU/mL).  Treatment with FOS and intravitreal therapy initiated with addition of CMVIG.  Repeat resistance testing identified additional *UL97* mutation (M460I).  Despite initial virologic response, complete suppression remained difficult due to fluctuating renal function, which affected drug dosing.  Control of retinitis was achieved with combination IV and intravitreal therapy.  At last follow-up, management of persistent low-level viremia remained ongoing. |
| **Key discussion points** | Persistent low-level viremia during prophylaxis warrants ongoing monitoring for potential dosing adjustments and/or resistance testing.  Proper coordination of care and delegation required to ensure timely review of test results and clinical follow-up.  Infectious disease consultation recommended for complicated CMV cases. |
| Abbreviations: CMV, cytomegalovirus; CMVIG, cytomegalovirus immunoglobulin; D+/R-; donor seropositive/recipient seronegative; eGFR, estimated glomerular filtration rate; FOS, foscarnet; GCV, ganciclovir; IV, intravenous; VGCV, valganciclovir. | |

| **CASE 1-2.** Sequential antiviral resistance in kidney transplant recipient *(Presented by Alaa Atamna)* | |
| --- | --- |
| **Patient history** | 51-year-old male with kidney transplant from living donor for end-stage renal disease due to focal segmental glomerulosclerosis.  CMV serostatus D+/R-.  Immunosuppression: prednisone, tacrolimus, mycophenolate.  CMV prophylaxis: VGCV 450 mg/day (institutional protocol for all kidney recipients regardless of risk status). |
| **Clinical course** | 5 months post-transplant, CMV syndrome developed (fatigue, leukopenia, elevated liver enzymes), with viral load of 120,000 IU/mL despite ongoing prophylaxis.  GCV initiated and immunosuppression reduced; viral load increased to 4 million IU/mL. Resistance testing confirmed GCV resistance (*UL97* mutation).  FOS initiated temporarily during MBV procurement.  MBV initiated, leading to initial response (Week 4 viral load 40,000 IU/mL), then rebound (100,000 IU/mL at Week 6). Resistance testing revealed MBV resistance (*UL97* T409M mutation).  FOS restarted, leading to undetectable viral load.  LET secondary prophylaxis initiated, then CMVIG added for persistent low-level viremia (~3000 IU/mL) despite normal IgG levels, resulting in undetectable viral load after first dose.  At last follow-up, patient remained on surveillance with CMV controlled |
| **Key discussion points** | Potential rapid development of MBV resistance, particularly with high viral loads, requires close monitoring.  Prophylaxis protocols deviating from risk-stratified guidelines (i.e., low dose) may contribute to breakthrough infection.  CMVIG may provide benefit as adjunctive therapy even with normal IgG levels. |
| Abbreviations: CMV, cytomegalovirus; CMVIG, cytomegalovirus immunoglobulin; D+/R-; donor seropositive/recipient seronegative; FOS, foscarnet; GCV, ganciclovir, IgG, immunoglobulin G; LET, letermovir; MBV, maribavir; VGCV, valganciclovir. | |

| **CASE 1-3.** Compartmentalized resistance in lung transplant recipient *(Presented by Renato Pascale)* | |
| --- | --- |
| **Patient history** | 57-year-old male with lung transplant for idiopathic pulmonary fibrosis.  CMV serostatus D+/R-.  Postoperative course complicated by renal impairment requiring dialysis.  Due to renal dysfunction, pre-emptive monitoring strategy selected over guideline-recommended prophylaxis. |
| **Clinical course** | CMV viremia developed as anticipated with pre-emptive approach; resolved with VGCV treatment.  Prophylaxis initiated after improvement of renal function but caused significant leukopenia, prompting discontinuation of VGCV and MMF.  CMV reactivated and progressed to CMV pneumonia (viral load 7 million IU/mL in bronchoalveolar lavage).  GCV initiated leading to partial response; CMVIG added leading to initial improvement followed by viral rebound.  Resistance testing revealed compartmentalized resistance: *UL97* mutation (L595S) in bronchoalveolar lavage but not in blood.  Switched from GCV to FOS with continued CMVIG; pneumonia resolved.  LET considered but denied by institution (off-label use for lung transplant not permitted).  At 5-month follow-up, CMV QuantiFERON test was positive, indicating immune reconstitution.  At last follow-up, patient remained on pre-emptive monitoring. |
| **Key discussion points** | Resistance testing should include site-specific samples when tissue-invasive disease is suspected, as compartmentalized resistance may not be detected in blood samples.  Compartmentalized resistance can occur with varying resistance profiles in different body sites.  Adoption of newer, guideline-recommended agents may be limited by institutional policies, e.g., restriction to approved indications. |
| Abbreviations: CMV, cytomegalovirus; CMVIG, cytomegalovirus immunoglobulin; D+/R-; donor seropositive/recipient seronegative; FOS, foscarnet; GCV, ganciclovir, LET, letermovir; MMF, mycophenolate mofetil; VGCV, valganciclovir. | |

| **CASE 1-4.** CMV management in heart transplant recipient with rare metabolic disorder  *(Presented by Isabell Just-Lauer)* | |
| --- | --- |
| **Patient history** | 28-year-old male with Kearns-Sayre syndrome presenting with ophthalmoplegia, hearing impairment, AV block (CRT in place), and mild ataxia.  Underwent heart transplant for severe heart failure.  CMV serostatus D+/R-.  Immunosuppression: tacrolimus, prednisolone, MMF.  CMV prophylaxis: VGCV. |
| **Clinical course** | Recurrent lactatemia (requiring dialysis) and fluctuating renal function made oral VGCV dosing unreliable; switched to GCV with therapeutic drug monitoring.  Viral load increased to >450,000 copies/mL over 2 weeks while achieving target drug levels.  Neurological symptoms developed (fluctuating vigilance, seizure, aspiration) requiring re-intubation. Concurrent development of DSA.  Workup including MRI and CSF negative for CMV CNS disease; resistance testing showed wild-type CMV.  Given risk of lactic acidosis with standard antivirals in mitochondrial disease, metabolic specialists advised CMVIG as “least worrisome” option.  GCV continued with addition of CMVIG.  MMF stopped and IVIG added for DSA management, during which viral load declined and neurological status cleared (DSA resolved).  Subsequent development of rejection, which responded to steroid pulses.  At last follow-up, CMV viremia had resolved and patient had been discharged to rehabilitation. |
| **Key discussion points** | Underlying metabolic or mitochondrial disorders may preclude safe use of standard antivirals.  CMVIG should be considered when patient-specific factors limit first-line options.  Multidisciplinary input (e.g., metabolic specialists) valuable in complex cases. |
| Abbreviations: AV, atrioventricular; CMV, cytomegalovirus; CMVIG, cytomegalovirus immunoglobulin; CNS, central nervous system; CRT, cardiac resynchronization therapy; CSF, cerebrospinal fluid; D+/R-; donor seropositive/recipient seronegative; DSA, donor-specific antibodies; GCV, ganciclovir; IVIG, intravenous immunoglobulin; MMF, mycophenolate mofetil; MRI, magnetic resonance imaging; VGCV, valganciclovir. | |

| **CASE 1-5.** CMV reactivation with competing clinical priorities in allo-HSCT *(Presented by Juliane Lohmeyer)* | |
| --- | --- |
| **Patient history** | 29-year-old female with AML and adverse-risk mutations (*p53, USAF1*) underwent allo-HSCT from a matched unrelated donor following relapse.  CMV serostatus D+/R+.  Pre-transplant prophylaxis for GVHD: ATG (conditioning), mycophenolate through day 45, tacrolimus through day 90 (early discontinuation planned due to high relapse risk).  Pre-transplant prophylaxis for CMV: LET through day 100. |
| **Clinical course** | Following LET discontinuation, acute grade 2-3 GVHD of the skin developed; treated with topical steroids and prednisolone.  Tacrolimus stopped per plan due to relapse risk, but restarted when liver and oral mucosal GVHD developed; CMV reactivation occurred.  Hepatitis and diarrhea with unclear etiology developed (GVHD vs. CMV); prednisolone restarted for presumed GVHD with Cytotect added for CMV.  VGCV initiated at renal-adjusted dosing, which was increased due to rising viral load; rebound occurred despite treatment.  MBV initiated for refractory CMV, which was managed on an outpatient basis due to patient circumstances (young mother with small children); viral load undetectable within 10 days.  GVHD resolved; patient weaned off prednisolone and tacrolimus.  At last follow-up, patient was off immunosuppression and in complete remission from AML. |
| **Key discussion points** | Symptom overlap between CMV and other conditions (in this case, GVHD) may pose challenges and require careful differentiation.  Competing clinical priorities and patient-specific circumstances (including preference for outpatient management) may require individualized decision-making that balances guideline recommendations against real-world constraints. |
| Abbreviations: allo-HSCT, allogeneic hematopoietic stem cell transplantation; AML, acute myeloid leukemia; ATG, antithymocyte globulin; CMV, cytomegalovirus; D+/R+; donor seropositive/recipient seropositive; GVHD, graft-versus-host disease; LET, letermovir, MBV, maribavir; VGCV, valganciclovir. | |

**Supplement 2. Additional expanded patient cases**

| **CASE 2-1.** CMV in kidney transplant recipient receiving complement inhibitor therapy  *(Presented by Nithya Krishnan)* | |
| --- | --- |
| **Patient history** | 26-year-old male with end-stage renal disease due to C3 glomerulonephritis (C3GN; a complement-mediated disease affecting 1-2 cases per million population).  Living donor kidney transplant via paired kidney exchange.  CMV serostatus D+/R-.  Immunosuppression: steroids, tacrolimus, mycophenolate.  CMV prophylaxis: VGCV.  Postoperative course complicated by hematoma requiring re-exploration. |
| **Clinical course** | Three weeks post-transplant, creatinine increased; biopsy confirmed C3GN recurrence.  Compassionate use of iptacopan (C3 convertase inhibitor) obtained after prolonged approval process.  Multiple complications ensued: acute glaucoma, retinal hemorrhage (steroid-related), atypical pneumonia requiring prolonged hospitalization.  Severe CMV infection developed with viral load reaching 1.65 million copies (900,000 IU/mL).  MMF and iptacopan discontinued; GCV and CMVIG initiated.  CMV declined to 35,000 copies (21,000 IU/mL).  Transplant ultimately failed; patient returned to dialysis.  Re-transplantation being considered with questions regarding preventive strategy. |
| **Key discussion points** | Whether complement inhibition increases CMV risk remains uncertain; audience polling revealed divided opinions.  Low C3 levels may predispose to CMV, suggesting complement inhibitors might theoretically be protective rather than harmful. Primary concern with complement inhibitors has traditionally been encapsulated bacteria rather than viral infections.  Case illustrates complexity of managing CMV with novel immunomodulatory agents not addressed in current guidelines. |
| Abbreviations: CMV, cytomegalovirus; CMVIG, cytomegalovirus immunoglobulin; D+/R-; donor seropositive/recipient seronegative; GCV, ganciclovir; MMF, mycophenolate mofetil; VGCV; valganciclovir. | |

| **CASE 2-2.** CMV encephalitis following CAR-T and bispecific antibody therapy  *(Presented by Yusri Taha)* | |
| --- | --- |
| **Patient history** | Male patient with 30-year history of follicular lymphoma (diagnosed 1996).  Multiple relapses over two decades, each successfully treated.  2020: Transformation to diffuse large B-cell lymphoma; autologous transplant with 3-year disease-free survival.  2023: Relapse treated with CAR-T.  Subsequent relapse treated with bispecific T-cell engager (BiTE) antibody therapy. |
| **Clinical course** | Approximately 6 months after BiTE therapy, CMV colitis developed.  Standard antiviral therapy initiated.  Progressed to CMV encephalitis with cognitive decline and gait disturbance.  MRI: enhancement in inferior basal ganglia, dorsal medulla, and proximal cord with evidence of hemorrhage.  Combined therapy with GCV and FOS employed.  Poor outcome despite aggressive treatment. |
| **Key discussion points** | End-organ CMV disease outcomes remain poor, highlighting the need for prevention.  Novel cellular therapies (CAR-T, BiTEs) are risk factors for CMV; cumulative immunosuppression from sequential treatments not well understood.  CMV-CMI monitoring may help stratify risk in these emerging populations, although not performed in this case.  Need for antivirals with better CNS penetration remains unmet. |
| Abbreviations: CAR-T, chimeric antigen receptor T-cell therapy; CMI, cell-mediated immunity; CMV, cytomegalovirus; CNS, central nervous system; FOS, foscarnet; GCV, ganciclovir; MRI, magnetic resonance imaging; VGCV; valganciclovir. | |

| **CASE 2-3.** Recurrent CMV in pediatric HSCT recipient with severe combined immunodeficiency  *(Presented by Julie Messiaen)* | |
| --- | --- |
| **Patient history** | 8-month-old male with severe combined immunodeficiency (HLA-DR deficiency due to homozygous *RFXANK* gene deletion).  Consanguineous parents.  At diagnosis: respiratory panel positive for RSV, enterovirus, CMV, parechovirus, coronavirus, rhinovirus, and *Pneumocystis jirovecii*.  Haploidentical HSCT from mother (no matched donor available).  CMV serostatus: D+/R+. |
| **Clinical course** | GCV initiated at diagnosis; CMV initially controlled around transplant.  Shortly post-HSCT, CMV viral load increased despite treatment.  CMVIG added; resistance testing performed (negative).  CMV copies continued rising despite dual therapy.  Switched from GCV to FOS with CMVIG continuation.  Gradual CMV control achieved; eventually transitioned to VGCV.  After 6 months’ hospitalization, patient discharged home.  Weeks later, readmitted for CMV reactivation with concurrent anemia. |
| **Key discussion points** | Pediatric HSCT presents unique challenges, including developmental pharmacology considerations and prolonged viral clearance times.  Haploidentical D+/R+ transplant setting represents very high-risk scenario.  Sequential therapy approaches may be necessary when initial treatment fails despite appropriate dosing.  Distinguishing between treatment failure, resistance, and immune reconstitution delays requires careful assessment.  Limited pediatric-specific data in guidelines necessitates extrapolation from adult studies. |
| Abbreviations: CMV, cytomegalovirus; CMVIG, cytomegalovirus immunoglobulin; D+/R+; donor seropositive/recipient seropositive; FOS, foscarnet; GCV, ganciclovir; HLA-DR, human leukocyte antigen DR isotype; HSCT, hematopoietic stem cell transplantation; RSV, respiratory syncytial virus; VGCV; valganciclovir. | |
