# Supplementary material for: The Troll Is Weakened but Not yet Defeated: An Update on Cytomegalovirus Management in Transplantation From the International CMV Symposium 2025
Source: Transpl Infect Dis. 2026 May 5;28(3):e70223. doi: 10.1111/tid.70223 (PMC13262554; doi:10.1111/tid.70223)
Supplement: Supplementary file 2 — Visual Abstract. [file TID-28-e70223-s001.pdf]

"The troll is weakened but not yet defeated": An update on cytomegalovirus management in transplantation from the International CMV Symposium 2025

@TheTxIDJournal @KottonNelson

Kotton CN *et al*. ***Transplant Infectious Diseases***. 2026.

2025 GUIDELINES UPDATES

- Letermovir prophylaxis expanded to high-risk SOT recipients
- Maribavir recommended for refractory/resistant CMV
- CMV-CMI testing to personalize management for R^+ kidney recipients
- Prophylaxis vs preemptive strategies refined

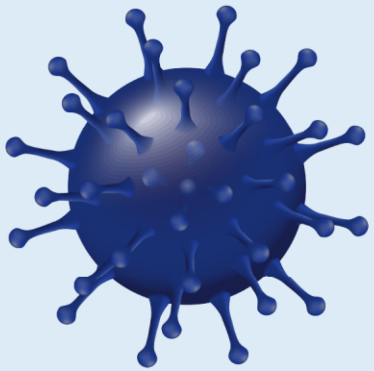

IMPLEMENTATION CHALLENGES

- Monitoring infrastructure varies by setting
- Cost and access barriers limit novel therapy adoption
- Regional and institutional variation in treatment protocols
- Individual patient factors require clinical judgment

Risk-stratified prevention • Patient-centered decision making • SOT + HSCT collaboration
